# Cognitive Outcomes for Congenital Hypothyroid and Healthy Children: A Comparative Study

**Published:** 2014

**Authors:** Mahtab ORDOOEI, Hadi MOTTAGHIPISHEH, Razieh FALLAH, Azar RABIEE

**Affiliations:** 1 Department of Pediatrics, Shahid Sadoughi University of Medical Sciences, Yazd, Iran; 2 Growth Disorders of Children Research Center, Department of Pediatrics, Shahid Sadoughi University of Medical Sciences, Yazd, Iran; 3 Shahid Sadoughi University of Medical Sciences, Yazd, Iran

**Keywords:** Congenital hypothyroidism, Cognitive assessment, Wechsler scale, Intelligent Quotient, Neonatal screening

## Abstract

**Objective:**

Early diagnosis and treatment of congenital hypothyroidism (CH) and the prevention of developmental retardation is the main goal of public health national screening programs. This study compares the cognitive ability of children with CH diagnosed by neonatal screening with a healthy control group (2007) in Yazd, Iran.

**Materials & Methods:**

In a case-controlled study, the intelligent quotient (IQ) of 40 five-year-old children with early treated CH and good compliance were evaluated by the Wechsler preschool and primary scale of intelligent test and compared to 40 healthy age and gender matched children as controls.

**Results:**

22 boys (55%) and 18 girls (45%) in both groups were evaluated. In children with CH, 19 (47.5%) and 21 (52.5%) persons had transient and permanent hypothyroidism, respectively.

Range of TSH and T4 level at the onset of diagnosis were 11.41–81 mu/l and 1.50–14.20 μg/dl, respectively.

The intelligence levels of all children with CH were within the average or normal range and IQs ranged from 91–108.

Children with CH had lower full-scale IQs (107.25 ± 2. 9 versus 110.50 ± 2.66, p=0.001), verbal IQ (106.95 ± 3.5 versus 109.90 ± 3.44, P-value=0.001) and performance IQ (106.3 ± 3.68 versus 108.87 ± 3.70) than the control group.

However, no statistically significant differences were observed for mean IQ scores in permanent and transient CH.

**Conclusion:**

Children with CH who had early treatment and good compliance had normal cognitive abilities, but may have a decreased IQ relative to the healthy control group.

## Introduction

Congenital hypothyroidism (CH) is the most common congenital endocrine disorder for children and it is one of the most important preventable causes of developmental retardation. The majority of neonates with CH is asymptomatic at birth and can be identified through neonatal screening programs. The incidence of congenital hypothyroidism based on screening tests has been estimated to be 1/3000 worldwide. Levothyroxine treatment with proper dosage should be started in the first weeks of life and continued at least up to 3 years of age. (1)

The nationwide neonatal CH screening programs were started in Iran by the Ministry of Health State Welfare Organization in 2005. The screening program used the measurement of TSH in filtered paper blood spots within 3–5 days after birth. The prevalence of CH in Iran is significantly higher than its prevalence worldwide (2-6).

Neurodevelopmental outcomes of CH children have been evaluated in different countries by different developmental assessment tests, which have debatable results. Mild impairments in cognitive and language abilities, poorer motor skills, speech and learning problems, lower intelligent quotient (IQ), incoordination, hypotonia or hypertonia, and short attention spans may occur in neonates with CH even with a timely diagnosis and proper treatment (1, 7, 8).

In some studies, no significant difference was seen in the IQs of children with CH and in normal healthy children (9, 10). However, in a study in Rotterdam, The Netherlands, over treated CH patients who were 5–7 years of age had higher global IQ scores than the control children did (10).

Minor and mild developmental disabilities in children with CH may not be detected in infancy, in early childhood, and are recognized by those with developmental delays before school entry, instead of waiting for more harsh problems that may arise later on, could aid to halt unnecessary problems in them and for their parents, and assessment of these children in preschool age might be more useful. A few studies compared developmental outcome of children with CH to matched controls.

This study evaluates and compares developmental outcomes and cognitive abilities of children with congenital hypothyroidism diagnosed by neonatal screening with a healthy control group of children in Yazd, Iran.

## Materials & Methods

In a case-control study, the cognitive functions for all singleton, term (gestational age = 37–42 weeks) neonates with congenital hypothyroidism that was diagnosed by neonatal screening in the city of Yazd, Iran in 2007 and for whom levothyroxine treatment in an adequate dosage was started in a timely manner (at less than one month of age), had good compliance, and had regular follow ups were assessed at the age of 5.

The control group consisted of 40 singleton, term, five-year-old healthy gender and socioeconomic status matched control children who were referred for vaccination to the primary health care center of Niko poor in 2012.

Multiple pregnancies, severe neonatal jaundice, and exchange transfusion, preterm neonates, severe asphyxia, NICU admission, children with major congenital malformations, chromosomal abnormalities, and genetic syndromes were excluded. 

Phone numbers, TSH, and T4 level at diagnosis, age at the onset of treatment, and type of CH (permanent or transient) for all children with CH who were diagnosed by neonatal screening in 2007 were found via records of the City Health Center of Yazd. 

Then, we called them and the importance of cognitive assessment and early detection of developmental delays was explained to the parents of CH patients. The parents were asked to take part in the study and bring their children into the Pediatric Endocrinology Clinic of Shahid Sadoughi University of Medical Sciences (Yazd, Iran) for cognitive assessment by a trained pediatric resident.

All children with CH were euthyroid at the time of intellectual and cognitive testing. Cognitive ability of children was assessed by the Persian version of the Wechsler Preschool and Primary Scale of Intelligent (WPPSI). Patient performance on the 11 subtests and three IQ scores were derived as follows: verbal IQ (VIQ), performance IQ (PIQ), and full-scale IQ (FSIQ) measuring general intellectual ability. In the normative population, each IQ score has a mean of 100 and a standard deviation of 15. The subtest scaled scores have a mean of 10 and a standard deviation of 3. For Quotient and Composite scores as follows: below 70 is Extremely Low; 70–79 is borderline, 80–89 is Low Average, 90–109 is Average, 110–119 is High Average, and 120–129 is Superior (11).

The data were analyzed with SPSS (ver 17). A Chi- square test or Fisher exact test was used for data analysis of qualitative variables. Mean values were compared for the two groups by an independent t-test and in three groups by the ANOVA test. 

Differences were considered significant when P-values were less than 0.05. 

The study was approved by the Ethics Committee of Shahid Sadoughi University of Medical Sciences (Yazd, Iran).

## Results

The parents of five children with CH refused to take part in the research. Finally, 40 five-year-old children with CH including 22 boys (55%) and 18 girls (45%) were evaluated and compared with 40 healthy age, gender, socioeconomic status, and race matched control children. In children with CH, 19 (47.5%) and 21 (52.5%) persons had transient and permanent hypothyroidism, respectively.

TSH levels at diagnosis were in the range of 11.41–81 mu/l with mean level of 19.53 ± 17.80 mu/l. T4 levels at the onset of treatment were in the range of 1.50–14.20 μg/dl with mean level of 6.50 ± 2.59 μg/dl. 

The intelligence levels of all children with CH were within the average or normal range and ranged from 91–108.

Table 1 presents a comparison of mean IQ scores in all cognitive domains for both groups and is indicative of statistically significant lower mean verbal IQ, performance IQ, and full-scale IQ scores in CH children. However, no statistically significant differences were seen for the mean of all IQ scores in permanent and transient CH.

Table 1 shows a comparison of mean IQ scores in all cognitive domains in children with CH based on T4 level and age at the onset of treatment; and indicates no observed statistically significant differences for these variables.

Six children had a TSH level greater than 6 mu/l during at follow up and the mean full-scale IQ was lower in these children (101.02 ± 3. 1 versus 112.78 ± 1.98; p=0.01).

## Discussion

In the present study, WPPSI was used for the evaluation of cognitive abilities of all neonates with CH detected by neonatal screening and the results show that these children had normal IQ scores, which agrees to other studies (9, 10, 12-17).

In this study, children with CH had a mean of verbal IQ, performance IQ, and full-scale IQ scores that were lower than for healthy socioeconomic status matched controls. In a study conducted in Zurich, Switzerland, children with CH showed lower full-scale IQ up to adolescents in spite of early treatment in high-doses and optimal substitution therapy in childhood (18). In other studies, CH children with early treatment had lower neurological scores (7); lower mean global IQ, verbal, and performance scores (19, 20, 21); had poorer academic performance in the early years (19); and intellectual deficits during childhood and adulthood (22).

In another Iranian study, the mean of global IQ of 18 children with transient CH at year 9 with the Raven and Bender-Gestalt tests was lower than the 19 matched control children but psychomotor performance was not significantly different in both groups (23).

In a study in Naples, Italy, 13 of 40 children with CH at 12-years of age showed subnormal IQ scores when compared with their siblings but the mean full IQ score was not significantly different in both groups (24). 

In the present study, T4 levels in the screening test had no effect on developmental outcomes for children with CH; this result complies with other studies (12, 13, 17, 22). Nevertheless, in some studies, neonates with severe hypothyroidism and very low serum T4 level (less than 2μg/dl) had lower cognitive scores and worse neurodevelopmental outcomes (7,19 ,20,21,24,25). 

In this study, the length of levothyroxine therapy had no effect on the cognitive abilities of children with CH, this agrees with other studies (12, 13, 20, 21, 22, 25). 

However, in a study in Paris, France, the IQ of infants who were treated after 21 days was lower than the control group (16).

Some persistent developmental deficits are likely related to genetic influences that exist regardless of therapy effectiveness (15).

Possible explanations for these discrepancies are as follows: age, race, geographic area, sample size, control group, and the genetics of children. 

**In conclusion, **based on our results, children with CH with early treatment (in the first month) and good compliance had normal cognitive and neurodevelopmental outcomes, but may have decreased IQ relative to a healthy control group. 

Early intellectual assessment of children with CH, the promotion of parental pedagogic attitude, and the encouragement of parents for the continuation of treatment and regular follow-ups should be done. 

**Table 1:** Comparison of Mean of Intelligent Quotients (IQ) Scores in All Cognitive Domains in Both Groups

Groups	Congenital hypothyroidism Mean ± SD				Healthy control Mean ± SD	P-value
Data	Permanent CH	Transient CH	P-value	Total CH		
Verbal-IQ	106.85 ± 3.03	107.05 ± 4.04	0.86	106.95 ± 3.5	109.90 ± 3.44	0.001
Performance-IQ	106.66 ± 3.66	105.89 ± 3.76	0.51	106.3 ± 3.68	108.87 ± 3.70	0.001
Full-IQ	107.33 ± 2.79	107.50 ± 3.07	0.85	107.25 ± 2.9	110.50 ± 2.66	0.001

**Table 2:** Comparison of Mean of IQ Scores in All Cognitive Domains in Children with Congenital Hypothyroidism Based on T4 Level and Age at the Onset of the Treatment

Full-IQ		Performance-IQ		Verbal-IQ		IQ Data	
P-value	Mean ± SD	P-value	Mean ±SD	P-value	Mean ± SD		
0.784	107± 1.91	0.839	107± 1.41	0.127	105.85 ± 3.38	< 4 µg/dl	T4 level at diagnose onset
	106.82± 2.48		106.23± 3.40		106.11 ± 3.37	4-7 µg/dl	
	107.81± 3.63		106.06± 4.68		108.31 ± 3.45	>7 µg/dl	
0.148	104± 3.33	0.709	105± 2.65	0.225	101.1± 3.12	<7 day	Age at the onset of the treatment
	106.95± 2.99		105.86± 3.57		107.04± 3.65	7-21 day	
	107.82± 2.74		106.94± 3.94		107.17± 3.18	22-30 day	
